# The Effect of Antioxidants on Dentin Bond Strength after Application of Common Endodontic Irrigants: A Systematic Review

**DOI:** 10.3390/ma16062260

**Published:** 2023-03-11

**Authors:** Regina Gascón, Leopoldo Forner, Carmen Llena

**Affiliations:** Department of Stomatology, Universitat de València, 46010 Valencia, Spain

**Keywords:** antioxidants, sodium hypochlorite, bond strength, adhesion, dentine, irrigants, systematic review

## Abstract

The purpose of this systematic review was to evaluate how an antioxidant treatment would influence the immediate bond strength of dental adhesives to endodontically treated dentine. Scientific evidence was researched in five databases using selected descriptors. Two independent reviewers conducted the investigation and assessed the data and methodological quality of the studies. Inclusion and exclusion criteria were specified for article selection; only studies in English and published between 2001 and 2022 were evaluated. A total of 18 studies were selected and analysed. According to most studies, the irrigation procedure and substrate preparation in endodontic therapy may affect the immediate bond strength. Antioxidants can improve bond strength and adhesion following the use of the most frequently used irrigants in root canal treatments. Therefore, antioxidant treatment can be considered a proper technique to enhance the bond strength of endodontically treated teeth.

## 1. Introduction

Success in endodontic treatment is based on the effective shaping and cleaning of root canals. It is a well-known fact that eliminating bacteria and biofilms from the root canal spaces is a difficult task in which the use of various instrumentation techniques alone is not effective [1]. Obtaining a favourable outcome depends on the eradication of microbes (present) in the root canal system and the prevention of reinfection with the use of mechanical procedures and chemical disinfecting products. The root canal is shaped under constant irrigation with the aim of removing not only the microbes/biofilms but the inflamed and necrotic tissue as well as other debris from the root-canal space that may interfere with the future sealing procedure [2]. Hence, infections of endodontic origin are mainly treated by mechanical procedures as well as chemical substances. All the functions required from an irrigant are not adequately recovered by a single irrigant solution. Optimal irrigation is based on the combination in a specific sequence of several irrigant solutions to obtain, in a predictable way, the goals of safe and effective irrigation [2,3]. 

Numerous irrigants have been recommended for eradicating bacteria and necrotic tissues of root canal infections. Sodium hypochlorite (NaOCl) has been widely used as an irrigant since its introduction in endodontics [4]. NaOCl has a disinfecting action as well as the ability to dissolve the pulpal remnants and organic components of dentine and predentine in non-instrumented surfaces [1]. NaOCl used in combination with other disinfectants such as Ethylenediaminetetraacetic acid (EDTA), citric acid, chlorhexidine (CHX), or alone represents the most used endodontic irrigant [3].

Nevertheless, the chemical irrigants (NaOCl, EDTA, and CHX) used during root canal treatments to provide gross debridement, lubrication, eradication of microbes, and decomposition of tissues, end up altering the chemical and mechanical characteristics of dentin, which affects its interaction with the restorative materials used for coronal sealing [5]. Radicals released in the process of the dissolution of tissues compete with vinyl-free radicals produced by the light curing of resins, which leaves incomplete the end of the chain and the polymerisation unfinished, thus compromising the bond strength of the adhesive system. In this process, the dentinal calcium and phosphate contents are reduced and the mechanical properties of dentin, such as the elastic modulus, bending strength, and hardness, are weakened. Thus, the micro-mechanical interactions between adhesive resins and the root canal dentin decrease after irrigation with sodium hypochlorite [6,7,8].

The quality of the permanent coronal restoration has a great influence on the general prognosis of endodontically treated teeth. In contemporary dental practice, the chance to restore such teeth with resin composite has increased as a result of the continuous revolution in adhesive system formulations [5]. On the other hand, these bond strengths compromised by NaOCl-treated dentin could affect the success of our treatment. 

To reverse the effects of these products on dentine and reduce the number of visits by our patients, the application of an antioxidant solution has been proposed prior to the adhesive procedure [9,10]. These antioxidants can interact with the NaOCl by-products, resulting in the neutralisation and reversal of the oxidising effect of the NaOCl-treated dentin surface [11] and hence, improve the success of our treatments. The antioxidants can interact with the residual oxygen released by irrigants, responsible for inhibiting the polymerisation of the resin composite, and, therefore, enhance the bond strength of the final restoration [9].

Thus, the aim of this study was to evaluate whether an antioxidant treatment would influence the immediate bond strength of dental adhesives to teeth exposed to any type of endodontic irrigant based on a systematic review.

## 2. Materials and Methods

This systematic review was described according to the Preferred Reporting Items for Systematic Reviews and Meta-Analysis (PRISMA Statement, Table S1) [12], and it was registered in OSF Registries (DOI 10.17605/OSF.IO/TDZPB).

### 2.1. Search Strategies

The literature research was carried out by two independent reviewers (R.G. and L.F.) who evaluated all the information published until 21 July 2022 and updated on 2 October 2022 considering unlimited publication date and no language restriction. The following databases were screened: Pubmed (Medline), Web of Science, Scopus, Embase, and Cochrane using the search strategy is described in Table 1. The asterisk (*) in Table 1 represents any group of characters, including no character, used at the endo of the root of the term.

### 2.2. Study Elegibility

The eligibility criteria were conducted according to the preferred reporting items for systematic reviews and meta-analysis (PRISMA) [13] guidelines and the Population, Intervention, Comparison and Outcome (PICO) design:

- P(opulation): in vitro clinical studies including human or animal enamel or dentine;
- I(ntervention): teeth in which an endodontic treatment was completed with sodium hypochlorite, EDTA, or chlorhexidine and that were subjected to an application of antioxidant agents and a following adhesive restoration;
- C(omparison): teeth in which an endodontic treatment but no application of antioxidant was completed before the adhesive restoration;
- O(utcome): bond strength tests, pull-out tests, or microscope observation.

Full-text manuscripts were reviewed and selected according to the following eligibility criteria: (1) in vitro studies that assessed the effect of the influence of antioxidants on the bond strength of adhesive systems to teeth that were endodontically treated using sodium hypochlorite, EDTA, chlorhexidine, or another irrigant solution; (2) studies contemplating a negative control group (without irrigant nor antioxidant); and (3) studies including analysis of shear, microshear, tensile, microtensile bond tests, or microscope evaluations produced by means of an objective, reproducible, and statistically supported method (if applicable). 

Parallelly, the following criteria were reasons for the exclusion of studies for qualitative synthesis: (1) manuscripts other than in English and Spanish (or another language that could be translated into these languages); (2) non-original articles (systematic review/narrative/umbrella, bibliometric/scientometric study); (3) studies and/or comparison of irrigants or antioxidants not used in the endodontic field; (4) analysis of parameters other than bond strength or adhesion (throughout mechanical or observational tests) of a resin composite in the coronal part of teeth.

### 2.3. Study Selection

Studies were, in the first place, recognized by title and abstract evaluation. Likewise, studies with deficient or inadequate information in the aforementioned sections to determine their relevance were included for detailed review. Full articles were obtained and thoroughly examined. The full-text papers were independently evaluated by two reviewers (R.G. and L.F.) who decided which of the studies were included in this review through discussion and consensus. 

### 2.4. Data Collection Process and Data Items

Relevant data were extracted using a standardised form in Excel 2019 MSO (Microsoft). Data included author; country; year; type of specimen (human or animal origin) and number; type, concentration, and time use of irrigant and the surface on which it was applied; type, concentration, and time of antioxidant application; type of bonding and steps; type of material used for obturation and steps; the statistical method used; and the bonding test used or microscopic observation. 

The specimens used in the study areas were subjected to a trial’s inclusion and exclusion criteria before their selection for the study. Once selected, the storage method was checked. 

One review author extracted the subsequent data from the included studies and arranged it in an Excel sheet, while the second author examined the extracted data. Disagreements were resolved by discussion and consensus between the two review authors; if no agreement could be reached, a third author was planned to make the final decision, but this was not necessary for any situation.

### 2.5. Assessment of Bias Risk and Quality of Included Studies

The methodological quality and risk of bias of each included study were evaluated by two reviewers based on the modified parameters established in previous systematic reviews of in vitro studies [14,15,16] and based on the Checklist for Reporting In Vitro Studies -CRIS Guidelines- [17].

The risk of bias in the articles was determined by the following parameters: teeth randomisation, use of sound teeth free of caries or restorations with similar dimensions, description of the sample size calculation, use of materials in accordance with the manufacturer’s instructions, procedures performed by a single operator, the blinding of the operator of the testing machine, and coefficient of variation [14]. If the authors reported the parameter, the study received a “YES” (Y) for that specific parameter. In the case of missing information, the parameter was assigned a “NO” (N). 

The coefficient of variation (CV) was determined for those articles that achieved shear and tensile strengths [18]. To determine the coefficient of variation parameter (CV, the ratio of the standard deviation to the mean), the results of each article were calculated and classified as low, medium, high, and very high [18]. Articles with low or medium CV outcomes were scored with a “YES,” whereas articles with high or very high CV were scored with a “NO.” The coefficient of variation was only achieved in those studies in which a bond strength test was accomplished.

Considering the before-mentioned parameters, articles that documented one to three data were normally classified as having a high risk of bias, four or five data with a medium risk of bias, and six or seven data with a low risk of bias [15]. Due to the fact that some of the studies were observational, the coefficient of variation was not applicable; hence, we decided to apply the risk of bias as a percentage, considering that it was more objective to compare the risk of bias among all studies. Thus, studies with a percentage below 60% were evaluated as having a high risk of bias, studies between 60% and 80% were evaluated as having a medium risk of bias, and studies with a percentage above 80% were evaluated as having a low risk of bias.

## 3. Results

### 3.1. Study Selection

**Figure 1:**
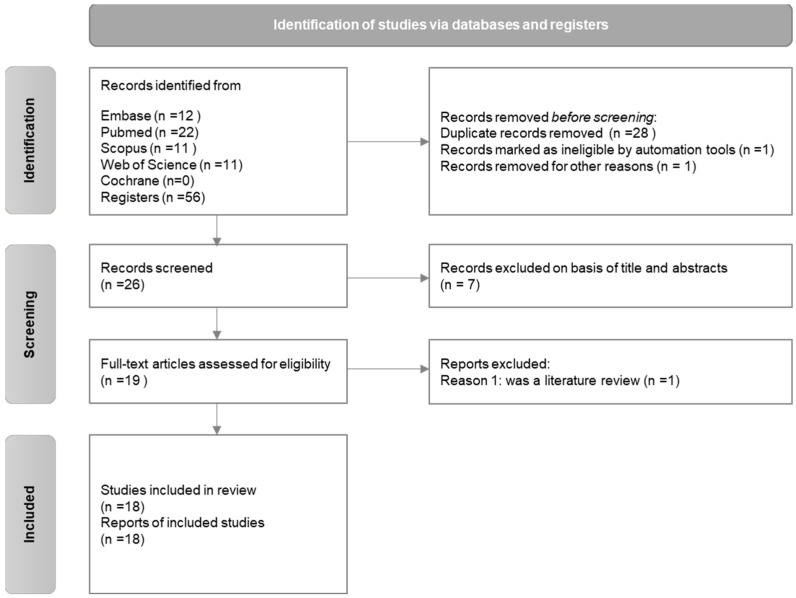
Flowchart showing the identification of studies via databases and registers [12].

A flow chart (Figure 1) is included to explain the reasons for the inclusion and exclusion of the examined articles. A total of five databases were consulted for information based on the eligibility criteria explained above (Table 1). In total, 56 studies were obtained according to the selected databases. A total of 28 duplicated records were removed, and 2 of them were excluded due to the impossibility of their reading. After the 26 articles were read, 7 additional studies were rejected because they did not fit in the original parameters (either they did not study adhesion or bond strength, or they did not use any of the specific irrigants or antioxidants). Subsequent to this screening, a total of 19 articles were selected, out of which, an additional 1 [14] had to be excluded because it was considered to be a review article. In the end, a total of 18 [19,20,21,22,23,24,25,26,27,28,29,30,31,32,33,34,35,36] full articles were included in our study. 

### 3.2. Study Characteristics

For each study, data were extracted and presented in Table 2.

- Epidemiological features: All the included articles were in vitro reports and published between 2001 and 2022 in English;
- Study locations: Six studies were fulfilled in India [19,20,21,22,23,24], three in Turkey [25,26,27], three in the USA [28,29,30], two in Brazil [31,32], two in China [33,34], one in Japan [35], and one in Iran [36].
- Specimens: All research was conducted in vitro. Most of the studies conducted tests on the extraction of human teeth [19,20,21,22,23,24,25,26,27,28,29,30,33,34,35,36] (except for two that studied bovine teeth [31,32];
- Storage Protocols: Teeth were stored in different chemical substances after extraction. Different storage conditions were presented such as sterile water [19], distilled water [21,22,23,25,31], or deionised water (Wang); in thymol at 0.2% [20,32] or 0.1% [35]; in a chloramine solution (in 0.5% for one week and subsequently in distilled water [26]; or in 0.2% [36]) and others used other combinations such as 0.02% sodium azide [34], 0.5% Chloramine T Trihydrate [29], and 0.9% NaCl containing 0.02% sodium azide [30]. Three studies did not indicate where the specimens were stored [24,27,28];
- Irrigation Protocols: A wide variety of irrigants was used. All studies tested the influence of NaOCl by itself or compared it with the effect of EDTA, chlorhexidine, povidone iodine, MTAD, and hydrogen peroxide. Seven authors studied the effect of NaOCl by itself in different concentrations at various times (5.25% NaOCl: 10 min [25,31] and 20 min [33]; 5% NaOCl: for 15–20 min [28]; and 5.25% NaOCl during 15–20 min [30]); less concentration and time (2.5% NaOCl, for 1 min [36], 3% NaOCl, for 2 min [22], 1% NaOCl, for 10 min) [23]; and a higher concentration (6% NaOCl for 20 min [29]);
- Some authors [19,20,23,32] combined the use of NaOCl with EDTA using a unique irrigation protocol for all groups of the study: Bansal et al. [19] used a total of 5 mL of 5.25% NaOCl followed by a rinse with 5 mL of 17% EDTA and final irrigation with 5 mL of 5.25% NaOCl; Bharti et al. [20] and Pimentel-Corrêa et al. [32] used the same irrigation protocol combining NaOCl and EDTA (5.25% of NaOCl for 30 min, followed by 17% EDTA for 3 min and a final rinse of 5.25% NaOCl for 1 min); and Nagpal et al. [23] used an irrigation protocol combining 1% NaOCl for 10 min and 17% EDTA for 1 min;
- Other authors compared the use of NaOCl with other irrigants: Bansal et al. [19] compared the use of NaOCl with Chlorhexidine (CHX) and Povidone Iodine (5,25% NaOCl for 1 min; 0.2% CHX for 1 min; and 5% povidone iodine); Dikmen et al. [26] compared NaOCl with EDTA and CHX (5.25% NaOCl for 30 s; 17% EDTA for 1 min followed by an application of 5.25% NaOCl for 30 s; 2% CHX for 5 min); Nassar et al. [35] compared NaOCl with CHX (10 mL 5% NaOCl for 10 min; 10 mL 5% NaOCl for 10 min and 5 mL 2% CHX); Pamir et al. [24] compared NaOCl with Hydrogen Peroxide (HP) (5% NaOCl; HP during 30 min of exposure); Sariyilmaz et al. [27] compared NaOCl with CHX (5.25% NaOCl for 30 min; 2% CHX for 30 min); and Shrestha et al. [34] compared NaOCl with MTAD and EDTA (using different groups of study with 1.3% NaOCl, 5.2% NaOCl, MTAD, 17% EDTA, 1.3% NaOCl + MTAD, or 1.3% NaOCl + MTAD);
- Bonding Techniques: Differences emerged when bonding was tested in the dentinal root or dentinal. When testing was performed on the coronal part of the tooth, the authors [19,20,22,23,24,25,26,30,32,33] always used a composite when testing the root, dual-cured cement [24], self-adhesive resin cement [36], AH Plus sealer [21], self-curing resin [28], Epiphany SE sealer [35], MTA [27], RealSeal SE [34], and three types of cements: Total etch technique (Variolink II); self-etching, self-adhesive dentin-bonding agent (Multilink and Clearfil Esthetic Cement EX); and self-etching and self-adhesive cements (SpeedCEM and Clearfil SA Cement) [29];
- Use of Antioxidant: The majority of the studies used 10% sodium ascorbate (SA) as an antioxidant agent, using it at different times [19,22,24,25,26,28,29,31,33,34,35], and Weston et al. [30] used 10% SA from 1 to 10 min. Bharti et al. [20] reduced it to 5% SA (5 min). The next most frequently used antioxidant was proanthocyanidin (PA, from grape seed extract) which was used in different concentrations such as 6.5% [21], 30% [22], 5%, 10%, and 15% [33]. Other antioxidants were used such as 10% rosmarinic acid [36], 10% hesperidin [36], 5% alpha-tocopherol [20,32], 5% sodium thiosulfate [20,27], and Bamboo Salt (BS) [21] and Quercetin [24] were also used;
- Testing Methods: When testing the bonding of these materials, some studies used microtensile or shear bond-strength tests [20,29,30,33,35]; others relied on SEM observation [19,22,23]; others used both [25,32,36]; and other authors used shear bond strength tests and observation under stereomicroscope [26,28,31], dye-penetration test under microscope [24], or Push-out bond strength tests [21,27,34].

**Table 2:** Summary of the data extracted from the articles reviewed.

Authors, Year and Country	Study Size	Surface to Which Material Was Bonded	Irrigant Used	Antioxidant	Adhesion or Bond Test Studied?	Material to Which Dentine Was Bonded to
Bansal et al., 2008 India [19]	116 human molars (8 groups of *n* = 12)	dentinal coronal (pulp chamber) surface	Sodium hypochlorite (NaOCl) 5.25% 1 min OR 0.2% Chlorhexidine (CHX) 1 min OR povidone iodine 5% 1 min	Sodium ascorbate (SA) 10% 1 min	Scanning Electron Microscopic Evaluation	Composite (Surefil (Dentsply) light cured for 40 s)
Bharti et al., 2021 India [20]	40 human incisors (5 groups of *n* = 8)	dentinal coronal surface	NaOCl 5.25% 30 min, AND Ethylenediaminetetraacetic acid (EDTA) 17% 3 min AND NaOCl 5.25% 1 min	SA 5% 5 min; alpha-tocopherol 5% 5 min; Na_2_S_2_O_3_ 5% 5 min (sodium thiosulfate)	Microtensile bond strength (μTBS)	Composite (Te-Econom plus Ivoclar Vivadent) light cured for 20 s
Celik et al., 2010 Turkey [25]	80 human molar teeth (*n* = 10)	dentinal coronal surface	NaOCl 5.25% for 10 min	SA 10% for 10 min	SEM observation; and Shear bond strengths	Composite Clearfil AP-X (Kuraray), polymerised for 40 s
Dikmen et al., 2018 Turkey [26]	60 human third molars (*n* = 5)	dentinal coronal surface	5.25% NaOCl for 30 s OR 17% EDTA 1 min + 5.25% NaOCl for 30 s OR 2% CHX for 5 min.	SA 10% 10 min	μTBS test and examined under a stereomicroscope	composite Filtek Z250
Furuse et al., 2014 Brazil [31]	30 bovine incisors (*n* = 5)	dentinal root surface	5.25% NaOCl 10 min	SA 10% 10 min.	μTBS and the failure modes were analysed through visual inspection with an 18× magnification under a stereomicroscope	dual-cured cement (RelyX ARC, 3M ESPE)
Khoroushi et al., 2013 Iran [36]	75 single-rooted human teeth (*n* = 15)	dentinal root surface	2.5% NaOCl 1 min	10% rosmarinic acid, 10% hesperidin, 10% SA 2 min	μTBS test and SEM examination	self-adhesive resin cement (Bifix SE, Voco Gmbh)
Kumar et al., 2019 India [21]	33 single rooted human teeth (*n* = 11)	dentinal root	5 mL of 5.25% NaOCl AND 5 mL of 17% EDTA AND 5 mL of 5.25% NaOCl	25% Bamboo Salt, 6.5% (proanthocyanidin) PA	Push-out bond strength testing and fractured samples were viewed under a stereomicroscope at 40× magnification	AH Plus sealer
Morris et al., 2001 USA [28]	56 human single-rooted teeth (*n* = 8)	dentinal root	5% NaOCl 15 to 20 min	10 mL of 10% SA 10 min	tensile bond strength and failed bonds were examined in a stereomicroscope at 15×	The self-curing resin (C & B Metabond) curing time, 10–15 min
Nagpal et al., 2007 India [22]	90 human premolars (*n* = 15)	dentinal coronal surface	3% NaOCl 2 min	SA 10% 1 min	Scanning Electron Microscopy	Composite Spectrum TPH (Dentsply Detrey) and light cured for 40 s
Nagpal et al., 2013 India [23]	66 human mandibular molars (*n* = 11)	dentinal coronal surface	1% NaOCl 10 min and 17% EDTA 1 min	30% PA for 1 min.	Scanning Electron Microscopy	composite resin Filtek Z250 (3M, ESPE)
Nassar et al., 2011 Japan [35]	50 extracted human molar (*n* = 10)	dentinal root	10 mL 5% NaOCl for 10 min OR 10 mL 5% NaOCl for 10 min AND 5 mL 2% CHX.	10 min 10 mL 10% SA	Shear Bond Strength Testing	Epiphany SE sealer was injected and light-cured for 40 s
Pamir et al., 2006 India [24]	70 single root human teeth (*n* = 10)	dentinal root	5% NaOCl, OR Hydrogen-peroxide	10 mL 10% SA 10 mL of Quercetin(Q) (does not specify concentration)	dye-penetration test under microscope	Resin composite [Ceram-x mono) and was cured for 40 s
Pimentel Corrêa et al., 2016 Brazil [32]	63 bovine incisors (*n* = 7)	dentinal coronal surface	5 mL 5.25% NaOCl for 30 min AND 5 mL 17% EDTA for 3 min AND NaOCl for 1 min.	5 mL 0.5% and 5% Sodium thiosulfate (Na_2_S_2_O_3_) solution for 1, 5, or 10 min	Microtensile Bond Strength and Scanning Electron Microscopic Analysis	1 mm of a resin composite (Filtek Z250, 3 M/ESPE)
Sariyilmaz et al., 2019 Turkey [27]	150 slices of root canal taken of canine human teeth (*n* = 30)	dentinal root	5.25% NaOCl solution for 30 min OR 2% CHX solution for 30 min	Sodium thiosulphate	Push-out bond strength tests	MTA
Shrestha et al., 2013 China [34]	100 single rooted human teeth (*n* = 10)	dentinal root	1.3% NaOCl OR 5.2% NaOCl OR MTAD OR 17% EDTA 1.3% NaOCl + MTAD OR 1.3% NaOCl + sodium ascorbate + MTAD OR 1.3% NaOCl + MTAD	SA 10% 1 min	Micro-Raman spectroscopic analysis and micro push-out bond test.	RealSeal SE
Stevens, 2014 USA [29]	70 human molars and premolars (*n* = 10)	dentinal root	6% NaOCl for 20 min	5 s in 1.2 mL 10% SA, 1 min in 2 mL 10% SA	Shear bond test	Three types of cements: Total etch technique (Variolink II); self-etching, self-adhesive dentin-bonding agent, f (Multilink and Clearfil Esthetic Cement EX); and self-etching and self-adhesive cements (SpeedCEM and Clearfil SA Cement)
Wang et al., 2019 China [33]	55 third molars (*n* = 5)	dentinal coronal surface	5.25% NaOCl for 20 min.	PA (5%, 10%, or 15%) for 1 min, 5 min, or 10 min.	Microtensile bond strength	composite resin (Clearfil AP-X light-cured 20 s
Weston et al., 2007 USA [30]	33 human single-rooted teeth (*n* = 6)	dentinal root	0.9% NaOCl OR 5.25% NaOCl 15–20 min per tooth	10% SA 10 min, 3 min and 1 min, 20%SA 1 min	Microtensile bond strength	C & B Metabond self-curing resin (curing time, 10–15 min).

### 3.3. Risk of Bias

The risk of bias in each study is shown in Table 3. All the gathered information is mentioned in the 2.5 Assessment of bias risk and quality of included studies (teeth randomisation, use of sound teeth, sample size description, use of materials according to the manufacturer’s instructions, single operators, blinding operator in the machine testing, and the coefficient of variation (as described above) [14,15,16]. Among the 18 studies, 7 were classified as high risk of bias, 9 studies as medium risk of bias, and 2 as low risk of bias.

### 3.4. Results of Individual Studies

All outcomes and statistical analysis evaluated in this systematic review are detailed in the table below (Table 4):

#### 3.4.1. Bond Strength Testing Method

When testing was performed on the coronal part of the tooth, five studies used the microtensile shear bond test [20,25,26,32,33]. The findings in these studies suggest that dentine is adversely affected by 5.25% NaOCl independently of the exposure time and the application of an antioxidant might have a reversal effect. Bharti et al. [20] demonstrated that the use of any of the three antioxidants (5% sodium ascorbate, 5% alpha-tocopherol, or 5% sodium thiosulfate) during 5 min reversed the effect of sodium hypochlorite when used with the total etch system (Te-Econom Bond). Celik et al. [25] studied the effect of 5.25% NaOCl in combination with three different types of adhesives: a two-step self-etch system (Clearfil SE Bond), two different one-step self-etch adhesives (Clearfil Tri-S Bond and Adper Prompt-L-Pop) as well as an etch and rinse adhesive (AdperSingle Bond 2). Here, the bond strength results were significantly influenced by the application of sodium ascorbate; however, this effect did not appear to be similar in all the adhesive systems used and may depend on their specific composition. Dikmen et al. [26] also studied the effect of NaOCl, NaOCl and EDTA, chlorhexidine, and sodium ascorbate on three different types of adhesive systems: two self-etching (Clearfil SE Bond (Kuraray Medical) and Xeno 3) and a total etch system (Single Bond). Dikmen et al. [26] concluded that the use of 10% sodium ascorbate after treating dentin with NaOCl significantly improved the bond strength of these adhesives. The application of CHX has no significant effect on the µTBS of the self-etch adhesives (Clearfil SE Bond and Xeno 3) but significantly lowered the bond strength of the total etch adhesive (Single Bond). For all adhesive systems, the EDTA + NaOCl-treated groups showed significantly decreased bond strength values than the control groups. Pimentel-Corrêa et al. [32] concluded that the reversal effects of sodium thiosulfate on the compromised bond strength in NaOCl/EDTA-treated dentin using the total etching adhesive system were found to be satisfactory when applied for 5 min, regardless of the concentration of 0.5% or 5%, and for only 1 min if using a 5% concentration. Nevertheless, the use of 5% Na_2_S_2_O_3_ for 10 min showed the best result. Wang et al. [33] only tested NaOCl and its effects on dentine’s bond strength. They used PA in different concentrations at various times and a two-step self-etch adhesive (Clearfil SE Bond). It was concluded that the treatment with 5% PA for more than 5 min or with 10% or 15% PA for more than 1 min improved the microtensile bond strength to NaOCl-treated dentine. The application of PA before an adhesive procedure may immediately restore the compromised bond strength of NaOCl-treated dentine.

Values changed and decreased when testing was performed on the radicular part of the tooth. Likewise, the results indicate that dentine is adversely affected by 5.25% NaOCl independently of the exposure time and that an antioxidant might help to reverse the bond strength. Furuse et al. [31] irrigated roots with a saline solution for 10 min (control), 5.25% NaOCl for 10 min, 5.25% NaOCl for 10 min, and 10% ascorbic acid following the use of 5.25% NaOCl for 10 min and then divided the groups based on the adhesive used: a three-step etch-and-rinse adhesive (Scotchbond Multi-Purpose Plus (SBMP)) or a one-step self-etching adhesive (Xeno III). All posts were cemented with the same dual-cured cement (RelyX ARC) and concluded that the decrease in bond strength after deproteinization is adhesive-dependent. The negative influence in bond strength of the self-etching (Xeno III) adhesive following deproteinization seems to be related to the oxidant effect of the NaOCl solution and the subsequent irrigation with SA may reverse the effect of the deproteinization. Khoroushi et al. [36] treated dentine with 2.5% NaOCl for 1 min and applied three different types of antioxidants (10% RA, 10% HPN, and 10% SA) for 2 min and concluded that NaOCl decreases the bond strength and the use of 10% SA or HPN for 2 min did significantly restore the compromised resin cement bond strength to NaOCl-treated dentin. Morris et al. [28] also studied the effect of NaOCl and an antioxidant (10% SA 10 min) on dentine, but in this case, they also added the effect of an RC-prep (a solution of 15% EDTA, 10% urea, HP and carbowax) and used a self-curing resin (C & B Metabond). They finished the endodontic surfaces after treatment with NaOCl or RC-Prep, decreased their bond strength, and encouraged the use of an antioxidant like 10% SA to restore this effect. Nassar et al. [35] included the use of 2% CHX in the normal irrigation with 5% NaOCl for 10 min and used SA for 10 min as an antioxidant. It was concluded that the use of CHX after an initial rinse of NaOCl is not enough to prevent the negative effect of NaOCl on the bond strength of the Epiphany SE sealer to dentin. A total of 10% SA might be the best method to counteract this detrimental effect of NaOCl and prevent the reaction between CHX and NaOCl; however, this would add an extra clinical step. In all groups, Stevens [29] used the same type of irrigation (6% NaOCl for 20 min) and only one type of antioxidant (10% SA) at different times (5 s and 1 min) and studied its effect on the five different cements tested. There were five brands of cements (three different types of adhesive systems): Type 1: Total etch technique (Variolink II); Type 2: self-etching, self-adhesive dentin-bonding agent, consisting of Multilink and Clearfil Esthetic Cement EX. (no acid needed); Type 3: self-etching and self-adhesive cements consisting of Speed CEM and Clearfil SA Cement (no adhesive and no acid needed). The cements that showed a decrease in bond strength on NaOCl-treated dentin were tested with two additional dentin treatment protocols in order to evaluate the potential reversal effects of treating the dentin with sodium ascorbate (5 s and 1 min) after NaOCl treatment. It was concluded that the total etch systems and systems with a separate dentin bonding agent were not negatively affected by previous NaOCl exposure. The self-etching, self-adhesive resin cements were negatively affected by NaOCl exposure. For those susceptible resin cements, a rinse with 10% sodium ascorbate provided an immediate restoration of at least 50% of the original bond strength. The efficacy of sodium ascorbate may vary among bonding systems. Finally, Weston et al. [30] demonstrated that 5.25% NaOCl irrigation produced a significant reduction in resin–dentin bond strengths (when using a self-curing resin (C & B Metabond) in dentinal root), but this can be reversed by treatment of at least 10% ascorbate for 1 min.

#### 3.4.2. Pull out Test

Only three studies [21,27,34] used push-out tests and all of them were conducted on the dentinal root part of the tooth. Independently of the type of irrigant and bonding technique, the values were similar to those with the bond strength test in the dentinal root part. Kumar et al. [21] concluded that the use of NaOCl as a final irrigant significantly decreases the dislocation resistance of AH Plus to root canal dentin and that the use of PA and BS as final irrigating solutions reversed the compromised pull-out bond strength of AH Plus to NaOCl-treated dentin. For instance, Sariyilmaz et al. [27], when testing with NaOCl and CHX and their inactivating agents (Sodium thiosulfate and L-alpha-lecithin, respectively), found out that these products had no significant adverse effect on the push-out bond strength of MTA after setting for 10 min. Shrestha et al. [34] concluded that the bond strength of RealSeal SE is adversely affected by NaOCl root canal irrigation; however, the use of 10% sodium ascorbate after NaOCl irrigation can reverse the compromised area. In conclusion, the authors recommended an application of sodium ascorbate between 5.2% NaOCl and EDTA to achieve the highest bond strength.

#### 3.4.3. Failure Mode Outcomes

Failure patterns were reported in six studies, where stereomicroscopy was used in four [21,26,28,31] and Scanning Electron Microscopy (SEM) in two [32,36], and both of them were used to determine the failure pattern and/or fractures in the interfaces. All six studies classified the failures as follows: (1) adhesive: deficiency between the resin/post and the adhesive system/luting cement or between the adhesive system/luting cement and the dentin; (2) cohesive: deficiency within the thickness of dentin or adhesive system; or (3) mixed deficiency associated with luting cement material covering 50% of the post diameter and the luting cement covering 50% of the post surface or if the fracture site continued from the adhesive system into either the resin composite or dentin. The adhesive/mixed failure pattern was predominant in all four studies [21,26,28,31]; however, Morris et al. [28] reported adhesive failures (between the adhesive system and the dentine) as the only cause of failure in the group in which the resin was placed after irrigation with 5% NaOCl without an antioxidant in between, and there were mixed patterns in the remaining groups. 

#### 3.4.4. Other Observation Tests

Two studies [19,24] implemented a dye-penetration test under microscope (×4) and recorded different types of results: 0—No evidence of dye penetration in the tooth/restoration interface; 1—Slight, only horizontal dye penetration; 2—Moderate, vertical Penetration less than half of the radicular composite restoration; 3—Extensive, vertical Penetration greater than half but less than the total of radicular composite restoration; 4—Gross, Vertical Penetration extending beyond the radicular composite restoration. In the study of Pamir et al.’s [24] study, in group 1 (NaOCl), the maximum number of teeth recorded a score of 4 which is the maximum dye penetration; in group 7 (Saline), the maximum number of teeth registered a score of 0 which is the minimum dye penetration. The use of SA after NaOCl (group 2) and H_2_O_2_ (group 5) irrigation significantly reduced the microleakage compared with their negative controls. In Bansal et al.’s study [19], extensive leakage was observed with both Prime & Bond NT (PBNT) and Xeno III after NaOCl irrigation (groups 1 and 4) and in PBNT after CHX and povidone-iodine irrigation (groups 2 and 3). Significantly less dye penetration was observed with Xeno III after CHX and povidone-iodine irrigation (groups 5 and 6). SA pretreatment after NaOCl allowed significantly less dye penetration in both PBNT and Xeno III groups (groups 7 and 8) than all other experimental and control groups except group 5 (CHX with Xeno III).

The studies of Nagpal et al. [22] and Nagpal et al. [23] analysed the degree of leakage penetration in two studies. The study of Nagpal et al. [22] found that both the conventional acid-etched groups and the acid-etched NaOCl-treated groups demonstrated extensive leakage. SA treatment of the deproteinized dentin significantly reduced the microleakage in comparison with the conventional acid-etched groups and acid-etched NaOCl-treated groups. Nagpal et al. [23] proved that the antioxidant (PA) treatment after NaOCl and EDTA irrigation significantly decreased microleakage in both adhesive systems.

Shrestha et al. [34] analysed their results with a micro-Raman spectrometer. Three spots on the sealer (2 µm next to the resin-dentin interface) were randomly chosen for Raman analysis and their mean was calculated. The Raman spectra were excited by a 785 nm laser line at a resolution of 1 cm. The laser beam was focused with an optical microscope at 50× magnification with approximately 4.94 mW laser power. All spectra (1500–1800 cm^−1^) were taken with one accumulation of 60 s exposure time and the value for a Degree of Conversion (DC) was calculated with a formula. Results were represented in a graphic display: the group with less degree of conversion was the one treated with NaOCl and the group with the most degree of conversion was the one treated with SA.

#### 3.4.5. Histological Outcomes

In six studies, SEM was used to evaluate the histological characteristics of dentin or the adhesive interface [19,22,23,25,32,36]. In 2010, Bansal et al. [19] detected the presence of gaps along the entire margin of the cavity in the NaOCl-treated groups showing poor adaptation at the resin–dentine interface. Sodium ascorbate application following the application of NaOCl with both adhesive systems revealed excellent adaptation of the resin composite to the cavity. Pretreatment of the cavity with CHX and povidone also showed the presence of a continuous gap and poor adaptation of the resin–dentine interface in both adhesive systems (PBNT and Xeno III), except for the group of CHX in combination with Xeno III that revealed an almost continuous margin and better adaptation of the resin composite to the dentine. Celik et al. [25] demonstrated that there was improved tubular penetration with lateral branches when sodium ascorbate was applied after the NaOCl treatment. Khoroushi et al. [36] reported that there is a much better adaptation between the resin cement and root dentin in groups control and those with the application of an antioxidant (SA and hesperidin)., The resin tags in these groups were apparently larger and more numerous. Nagpal et al. [22] reported that Prime & Bond NT showed better interfacial adaptation, deeper tubular penetration, and filled lateral tubular branches when used after the NaOCl/sodium ascorbate treatment. Nagpal et al. [23] stated that when PA treatment followed the conventional NaOCl and EDTA irrigation regimen, an excellent interfacial adaptation without gap was observed for both adhesives with pulpal dentin. Pimentel Corrêa et al. [32] revealed that adhesive failure was the most common fracture pattern observed regardless of the experimental condition.

## 4. Discussion

This systematic literature review was directed to gather and analyse the available evidence of in vitro studies on the effect of antioxidants of different kinds on dentin exposed to the effects of different types of irrigants and its following adhesive restoration.

Considering the quality evaluation in relation to the studies available, very few [20,26] scored a low risk of bias, and this exposes the quality of the evidence generated by this systematic review. Unfortunately, because of this lack of published research on low bias, the present study takes into account the medium and high risk of biased documents; hence, the results should be considered with caution. 

The purpose of this systematic review was to analyse the effects of antioxidants after the use of irrigants on dentine. According to most studies [19,20,21,22,23,24,25,26,27,28,30,31,32,33,34,35,36], antioxidants can improve bond strength following the use of the most frequently used irrigants in root canal treatments.

The use of antioxidants to improve the adhesive union of final restorations has increased recently [37]. Various antioxidants in different concentrations and times have been proposed in recent years [38,39,40,41,42,43,44,45,46]. Feiz et al. [37] showed in their systematic review that the adverse effect of bleaching agents on shear bond strength may be reversed by all antioxidants. In this systematic review, in which the effect of antioxidants on bleached dentine was analysed, it was also concluded that a one-week delay before using the restorative materials can be as effective as using antioxidants in the majority of cases. 

We found a wide variety of antioxidants among the queried studies. Sodium ascorbate (SA) was the most used in different concentrations (5%, 10%, 20%, 35%) [44,47,48,49], but oligomeric proanthocyanidin complexes (OPCs), present in grape seed extracts [40,42,43,49,50,51,52] and pomegranate peel extract [43,49], green tea [19,43,53,54,55,56,57], alpha-tocopherol [52], aloe vera [41,43,51], and licopene [40] were also used. The antioxidant considered to be the gold standard is SA and, in most studies, it is used by itself or in the comparison of its effect with other antioxidants [14].

The irrigation protocols also varied among studies. Irrigation with NaOCl between 2% and 5% could be observed in different exposure times as well as combinations with 17% EDTA, which makes it difficult to determine a conclusion, whereas the effect of the decrease on bond strength is due to a combination of products, exposure time, or its concentration. 

Several restoration strategies and different testing methods were used in this systematic review. If data were evaluated with the same techniques and methods, more consistent results could be obtained, providing assistance for the clinician with proof-based decision-making. 

Regarding restoration strategies, several studies focused on the dentine in the pulp chamber region (coronal dentine) where all of them tested the composite resin materials. These studies [19,20,22,23,25,32,33] combined bond strength with observational tests. Four out of seven studies [20,25,32,33] used a bond strength test to analyse the results combining it with or without observational tests, whereas three out of seven studies [19,22,23] only used observational tests. Regardless of the study conditions, all studies except one [29] concluded that the use of an antioxidant after being treated with NaOCl revealed an excellent adaptation of composite resins to dentinal walls in the observational tests and better parameters in the bond strength tests. Stevens et al. [29] considered that providing an adhesive strength with a minimum of 50% of the original bond strength with a rinse of 10% sodium ascorbate was not enough to consider it effective.

When testing was performed on the coronal part of the dentine, all studies used a composite resin for the restoration [19,20,22,23,25,26,32,33]. Another factor that could be analysed as a potential reason for the risk of bias is the type of adhesive system. There were different types of adhesive systems used to bond the composite resin which produced different results, but it looks like the deproteinization of the dentin due to NaOCl exposure equally affects all types of adhesives. However, antioxidants can restore the bond strength in all of them. There is no clear evidence about how the effect of other irrigants (such as CHX) would affect the bond strength of the diverse types of adhesive solutions. There is neither enough evidence about how the type of irrigant and the use of an antioxidant may be affected by the different adhesive systems available. More clear scientific evidence is required. Celik et al. [25] studied the effect of 5.25% NaOCl in combination with three different types of adhesives: a two-step self-etch system (Clearfil SE Bond), two different one-step self-etch adhesives (Clearfil Tri-S Bond and Adper Prompt-L-Pop) and an etch and rinse adhesive (AdperSingle Bond 2). Here, the application of sodium ascorbate significantly influenced the bond strength results, but this effect did not seem to be alike in all the adhesive systems utilized and may depend on their specific composition. Dikmen et al. [26] also studied three different types of adhesive systems: two self-etching (Clearfil SE Bond (Kuraray Medical) and Xeno 3) and a total etch system (Single Bond). Dikmen et al. [26] determined that the use of 10% sodium ascorbate after treating dentin with NaOCl improved, in a significant way, the bond strength of these adhesives, but when CHX is used as an irrigant, it has no significant effect on the µTBS of self-etch adhesives (Clearfil SE Bond and Xeno 3) but notoriously decreased the bond strength of the whole etch adhesive (Single Bond). Thus, the type of irrigant protocol might depend on the type of adhesive system used to restore the composite resin. Pimentel-Corrêa et al. [32] used a total etching adhesive system and found it to be acceptable when applied for 5 min, regardless of its concentration. Wang et al. [33] used a two-step self-etch adhesive (Clearfil SE Bond) and determined that the application of PA before an adhesive procedure may immediately restore the compromised bond strength of NaOCl-treated dentine. 

When testing is carried out in the root dentine, the restorative technique varies even more. Two studies [31,36] cemented posts to study its union. Furuse et al. [31] cemented posts with the same dual-cured cement (RelyX ARC) and two different types of adhesive solutions: a three-step etch-and-rinse adhesive (Scotchbond Multi-Purpose Plus (SBMP)) or a one-step self-etching adhesive (Xeno III). Furuse et al. [31] concluded the negative effect of deproteinization is adhesive-dependent and has an adverse effect only upon the self-etching adhesive, but it can be reversed by the application of an antioxidant (SA 10% 10 min). Khoroushi et al. [36] also cemented posts with a self-adhesive resin cement (Bifix SE, Voco) and concluded the self-adhesive is negatively affected by the deproteinization of the NaOCl but its effects can be restored with the use of an antioxidant during 2 min (SA, HPN, and RA, in this case). Kumar et al. [21] used an epoxy resin-based sealer, AH Plus, and determined that the use of NaOCl as a final irrigant significantly decreases the dislocation resistance of AH Plus to root canal dentin but that the use of an antioxidant (PA and BS) as final irrigating solution reverses the situation. Morris et al. [28] filled the root canal space with a self-curing resin (C & B Metabond) that saw its bond strength decrease after the irrigation of NaOCl but the antioxidants (SA) helped reverse the situation. Nassar et al. [35] injected an Epiphany SE sealer into the root canal and we can extract from their study that apparently, the use of CHX after an initial rinse of NaOCl is not sufficient to counteract the adverse effect of NaOCl on the bond strength of the Epiphany SE sealer to dentin. Moreover, they suggest that the application of SA might be the most appropriate method to reverse this detrimental effect of NaOCl and avoid the reaction between CHX and NaOCl. Pamir et al. [24] used a resin composite (Ceram-X mono) and observed higher leakage scores in NaOCl and HP groups that could be reverted with the application of an antioxidant (SA). Sariyilmaz et al. [27] used MTA and could not show that deproteinization negatively affected the union of MTA to dentine; however, the application of an antioxidant could help increase the bond strength of this material. Stevens [29] used five types of cements: a total etch technique (Variolink II); two self-etching, self-adhesive dentin-bonding agents, consisting of Multilink and Clearfil Esthetic Cement EX; and two self-etching and self-adhesive cements consisting of SpeedCEM and Clearfil SA Cement. The five resin cements used differed in their immediate shear bond strength to NaOCl-treated dentin. Previous NaOCl exposure did not affect in a negative way the total etch solutions and solutions with a separate dentin bonding agent. The self-etching, self-adhesive resin cements were negatively affected by NaOCl exposure but for those susceptible resin cements, a rinse of 10% sodium ascorbate improved the bond strength, but not up to values of the control group. Weston et al. [30] used a self-curing resin (C & B Metabond) and concluded it was sensitive to pretreatment with NaOCl, decreasing its bond strength values, but using an antioxidant irrigation improved its bond strength. 

When testing in the radicular part of the tooth, there are not two single studies that reproduce the same restorative conditions. Most of them conclude that NaOCl negatively affects the union of the restorative material [21,24,28,31,35,36] and that the use of an antioxidant [21,24,27,28,30,31,35,36] helps increase the union of the final restorative material to the radicular dentin. These results are supported by Baruwa et al.’s study [58] which suggests that the best method to increase the bond strength of posts in dentine would be a combination of NaOCl, citric acid, and final irrigation of CHX. These authors expose that a final rinse with CHX to improve the final bond strength should be convenient. The studies that compared different types of adhesive systems [29,31,35] concluded that the negative effect of deproteinization is adhesive-dependent and affects mostly self-adhesive systems. This could also be supported by Weston et al.’s study [30], which does not compare different types of adhesive systems by does observe that there is a decrease in the bond strength of its self-curing cement after the pretreatment with NaOCl.

## 5. Conclusions

Considering the limitations of this systematic review (only two studies included in the systematic review scored a low risk of bias and there is huge heterogeneity among the studies), the consequences for clinical practice included in the literature were the following:

1. The deproteinization caused by the NaOCl in irrigating protocols in endodontic procedures affects the immediate and long-term bond strength when using an adhesive system;
2. The decrease in bond strength and adhesion in dentin may be restored using an antioxidant agent;
3. The use of an antioxidant such as 10% sodium ascorbate may be established as a proper agent to enhance the bond strength of the endodontically treated teeth;
4. The negative effect of deproteinization is adhesive-dependent and affects mostly self-adhesive systems, but additional efforts will be required to determine which type of adhesive system would be of the best election.

## Figures and Tables

**Table 1:** Search terms used in databases.

Data Bases	Search Terms
Web of Science	(((ALL = (“sodium hypochlorite” OR edta OR chlorhexidine OR irrig*)) AND ALL= (endod*)) AND ALL= (adhes* OR bond*)) AND ALL (antiox*)
Pubmed (MEDLINE)	(“sodium hypochlorite” OR edta OR chlorhexidine OR irrig*) AND (endod*) AND (adhes* OR bond*) AND (antiox*)
Embase	(“sodium hypochlorite” OR edta OR chlorhexidine OR irrig*) AND (endod*) AND (adhes* OR bond*) AND (antiox*)
Cochrane	(“sodium hypochlorite” OR edta OR chlorhexidine OR irrig*) AND (endod*) AND (adhes* OR bond*) AND (antiox*) in All Text Topics: Dentistry and oral health and Dental caries
Scopus	(“sodium hypochlorite” OR edta OR chlorhexidine OR irrig*) AND (endod*) AND (adhes* OR bond*) AND (antiox*)

**Table 3:** Risk of bias in each study.

Authors	Teeth Randomisation	Use of Sound Teeth	Sample Size Description	Use of Materials according to the Manufacturer’s Instructions	Single Operator	Testing Machine Operator Blinding	Coefficient of Variation	Risk of Bias
Bansal et al. [19]	YES (Y)	Y	Y	Y	NO (N)	N	N.A. (not applicable)	MEDIUM
Bharti et al. [20]	Y	Y	Y	N	Y	Y	LOW: YES	LOW
Celik et al. [25]	Y	Y	Y	Y	N	N	LOW: YES	MEDIUM
Dikmen et al. [26]	Y	Y	Y	Y	N	Y	LOW: YES	LOW
Furuse et al. [31]	N	Y	N	Y	N	N	VERY HIGH: NO	HIGH
Khoroushi et al. [36]	Y	Y	Y	Y	N	N	VERY HIGH: NO	MEDIUM
Kumar et al. [21]	N	Y	Y	N	N	N	VERY HIGH: NO	HIGH
Morris et al. [28]	Y	Y	Y	Y	N	N	INTERMEDIATE: YES	MEDIUM
Nagpal et al. [22]	N	Y	Y	N	N	Y	N.A.	HIGH
Nagpal et al. [23]	Y	Y	Y	Y	N	N	N.A.	MEDIUM
Nassar et al. [35]	Y	Y	Y	N	N	N	LOW: YES	MEDIUM
Pamir et al. [24]	N	Y	Y	N	N	N	N.A.	HIGH
Pimentel-Corrêa et al. [32]	Y	N	N	N	N	N	LOW: YES	HIGH
Sariyilmaz et al. [27]	Y	N	Y	N	N	N	LOW: YES	HIGH
Shrestha et al. [34]	Y	N	Y	Y	N	N	LOW: YES	MEDIUM
Stevens et al. [29]	Y	Y	Y	Y	N	N	INTERMEDIATE: YES	MEDIUM
Wang et al. [33]	Y	Y	Y	N	N	N	N.A.	HIGH
Weston et al. [30]	Y	Y	Y	Y	N	N	INTERMEDIATE: YES	MEDIUM

**Table 4:** Summary statistic for each study.

Authors	Surface to Which Material Was Bonded	Testing Method	Results	Type of Statistical Analysis	Outcomes
Bansal et al., 2008 India [19]	dentinal coronal (pulp chamber) surface	SEM Observation		Scores were analysed with Kruskal–Wallis nonparametric analysis followed by Mann–Whitney U-test to evaluate differences among the experimental groups at a significance level of *p* = 0.05.	Effective
Bharti et al., 2021 India [20]	dentinal coronal surface	Microtensile bond strength (μTBS) test	Group I (Positive control): 5 mL of 5.25% NaOCl-30 min and 17% EDTA 3 min and 5.25% NaOCl 1 min 15.38 ± 1.22 Group II (Negative control): No irrigation and no antioxidant. 21.49 ± 1.48 Group III: As in group 1 + 5 mL of 5% SA 10 min 19.36 ± 1.39 Group IV: As in group I + 5 mL of 5% Alpha Tocopherol 10 min 17.90 ± 1.54 Group V: As in group I + 5 mL of 5% Na_2_S_2_O_3_ 10 min 22.38 ± 0.84	Under the normal distribution, the data were analysed by one-way ANOVA (*p* = 0.05). Comparison of Microtensile bond strength between the groups was explained by Mann–Whitney U test.	Effective
Celik et al., 2010 Turkey [25]	dentinal coronal surface	SEM observation; and Shear bond strengths	Group 1: 5.25% NaOCl for 10 min + Clearfil SE Bond (NaOCl/CSE) 21.15 ± 5.23 Group 2: 5.25% NaOCl for 10 min + Clearfil Tri-S Bond (NaOCl/CTS)21.37 ± 8.24 Group 3: 5.25% NaOCl for 10 min + Adper Prompt-L-Pop (NaOCl/APLP) 19.26 ± 5.10 Group 4: 5.25% NaOCl for 10 min + Adper Single Bond 2 (NaOCl/ASB2) 17.68 ± 5.11 Group 5: 5.25% NaOCl for 10 min + Sodium Ascorbate (10%)10 min + Clearfil SE Bond (ASC/CSE) 24.26 ± 6.03 Group 6: 5.25% NaOCl) for 10 min + Sodium Ascorbate (10%) 10 min + Clearfil Tri-S Bond (ASC/CTS) 27.37 ± 6.17 Group 7: 5.25% NaOCl for 10 min + Sodium Ascorbate (10%) 10 min + Adper Prompt-L-Pop (ASC/APLP) 32.37 ± 5.45 Group 8: 5.25% NaOCl for 10 min + Sodium Ascorbate (10%) 10 min + Adper Single Bond 2 (ASC/ASB2) 19.52 ± 4.02	The data were analysed by two-way ANOVA. Differences between the groups were analysed using a post hoc Tukey HSD test (*p* < 0.05).	Effective
Dikmen et al., 2018 Turkey [26]	dentinal coronal surface	μTBS test and stereomicroscope examination	Single Bond Group and Control: 37.2 ± 5.0; and NaOCl 18.9 ± 4.3; and NaOCl + EDTA 15.4 ± 3.9; and CHX 22.0 ± 4.5; and NaOCl + SA 29.0 ± 4.9. SE Bond Group and Control: 39.5 ± 5.2; and NaOCl 26.9 ± 6.5; and NaOCl + EDTA 22.0 ± 5.9; and CHX 39.8 ± 4.8; and NaOCl + SA 34.8 ± 5.2. Xeno 3 Group and Control: 20.7 ± 5.0; and NaOCl 21.1 ± 4.9; and NaOCl + EDTA 16.0 ± 5.1; and CHX 18.0 ± 4.7; and NaOCl + SA 23.2 ± 4.2.	The Kolmogorov–Smirnov test was used to assess for a normal data distribution. The mean bond strength data were statistically analysed by two-way analysis of variance (ANOVA) and one-way ANOVA. The level of statistical significance was set at *p* < 0.05.	Effective
Furuse et al., 2014 Brazil [31]	dentinal root surface	μTBS test and stereomicroscope examination	SBMP Group: Apical: Control 1.98 (1.35); NaOCl 1.14 (0.71); NaOCl + SA 2.86 (0.61) Middle: Control 5.30 (1.69); NaOCl 4.65 (1.44); NaOCl + SA 5.26 (0.93) Coronal Control 9.59 (3.29); NaOCl 6.73 (2.24); NaOCl + SA 7.35 (2.04) Xeno III Group: Apical: Control 5.04 (2.53); NaOCl 0.33 (0.10); NaOCl + SA 1.70 (0.54) Middle: Control 9.27 (2.86); NaOCl 0.91 (0.28); NaOCl + SA 4.68 (1.24) Coronal: Control 11.69 (5.02); NaOCl 3.87 (1.09); NaOCl + SA ± 10.09 (2.1)	The data were statistically analysed using three-way ANOVA. Multiple comparisons were made using Tukey’s test (α = 0.05).	Effective
Khoroushi et al., 2013 Iran [36]	dentinal root surface	μTBS test and SEM examination	1. Control-(irrigated with normal saline) 9.27 ± 3.19 2. Control+ (2.5% NaOCl 2 min) 6.71 ± 2.56 3. 2.5% NaOCl 2 min + RA 8.86 ± 3.02 4. 2.5% NaOCl 2 min + HPN 9.30 ± 2.49 5. 2.5% NaOCl 2 min + SA 10.03 ± 2.59	The data were analysed using the Kolmogorov-Smirnov test, one-way ANOVA, and Tukey’s HSD test (α = 0.05).	Effective
Kumar et al., 2019 India [21]	dentinal root	Push-out bond strength test and stereomicroscope examination	1. 5.25% NaOCl: coronal 0.34 ± 0.15; middle 0.68 ± 1.06; apical 0.53 ± 0.22 2. 6.5% PA: coronal 2.22 ± 0.73; middle 1.99 ± 0.33; apical 1.16 ± 0.17 3. 25% BS: coronal 1.65 ± 0.34; middle 1.84 ± 0.55; apical 1.42 ± 0.30	The PBS data were subjected to Kruskal–Wallis and Dunn’s post hoc test. The significance was set at *p* < 0.05.	Effective
Morris et al., 2001 USA [28]	dentinal root	μTBS test and stereomicroscope examination	G1. 0.9% NaCl: 23.6 ± 2 4.5 G2. 2 5% NaOCl: 7.72 ± 4.6 G3. RC-Prep (15% EDTA, 10% urea, HP and carbowax): 14.0 ± 4.6 G4. 0.9% NaCl + 10% SA 10 min: 25.9 ± 3.9 G5. 5% NaOCl + 10% SA 10 min: 27.7 ± 4.5 G6. 5% NaOCl + 10% SA 10 min: 30.6 ± 3.7 G7. RC-Prep + 10% SA 10 min: 21.1 ± 4.8	A two-way analysis of variance was performed using surface treatments. Multiple comparison post hoc tests were performed using Tukey’s honest significance difference test at α = 0.05.	Effective
Nagpal et al., 2007 India [22]	dentinal coronal surface	SEM observation		The results were analysed with Kruskal–Wallis non-parametric analysis followed by Mann–Whitney U test to evaluate differences among the experimental groups at a significance level of *p* = 0.05.	Effective
Nagpal et al., 2013 India [23]	dentinal coronal surface	SEM observation		Microleakage scores were statistically analysed by Kruskal–Wallis non-parametric analysis and Mann–Whitney U-tests at a significance level of *p* < 0.05.	Effective
Nassar et al., 2011 Japan [35]	dentinal root	μTBS Test	G I, control (deionized water) 0.29 ± 0.10 G II (NaOCl) 0.30 ± 0.12 G III (NaOCl/SA) 0.93 ± 0.13 G IV (NaOCl/CHX) 0.53 ± 0.11 G V (NaOCl/SA/CHX) 0.99 ± 0.14	One-way analysis of variance (ANOVA) was used to compare the mean shear bond strengths of the 5 groups, and the Tukey test was performed for post hoc comparisons. The significance level was set at 0.05.	Effective
Pamir et al., 2006 India [24]	dentinal root	dye-penetration test under microscope		Kruskal–Wallis test, Mann–Whitney U, and Wilcoxon W tests were used. The significance level was set at *p* < 0.05.	Effective
Pimentel Corrêa et al., 2016 Brazil [32]	dentinal coronal surface	μTBS test and stereomicroscope examination	Negative control 31.26 (6.81) Positive control 16.73 (8.24) (5.25% NaOCl 30 min + 17% EDTA 3 min) SA 0.5%/1 min 15.14 (3.52) SA 0.5%/5 min 25.12 (10.13) SA 0.5%/10 min 24.46 (8.54) SA 5%/1 min 23.64 (10.83) SA 5%/5 min 23.74 (8.01) SA 5%/10 min 31.98 (9.03) NaCl/10 min 15.33 (4.26)	The data were determined and analysed by 1-way analysis of variance and the Duncan test. The statistical significance level was set at *p* < 0.05	Effective
Sariyilmaz et al., 2019 Turkey [27]	dentinal root	Push-out bond strength tests	Control 8.67 ± 3.11 G 1 (NaOCl) 9.15 ± 3.10 G 2 (NaOCl—Sodium thiosulfate) 10.75 ± 2.83 G 3 (CHX) 8.25 ± 2.07 G 4 (CHX—L-alfa-lecithin) 7.49 ± 2.75	The normality of the push-out bond strength data distribution was confirmed with a Shapiro-Wilk test, and the data were analysed by one-way analysis of variance and Tukey multiple comparison tests with the level of significance set at 5%.	Effective
Shrestha et al., 2013 China [34]	dentinal root	micro-Raman spectroscopic analysis and micro push-out bond test.	G1. Distilled water 3.70 ± 0.844 G2. 1.3% NaOCl 8.88 ± 1.76 G3. 5.2% NaOCl 9.00 ± 1.90 G4. MTAD 9.28 ± 1.62 G5. 17% EDTA 8.71 ± 1.43 G6. 10% SA 4.03 ± 1.02 G7. 1.3% NaOCl/MTAD 4.05 ± 1.04 G8. 1.3% NaOCl/SA/MTAD 7.15 ± 1.56 G9. 5.2% NaOCl/17% EDTA 9.34 ± 1.83 G10. 5.2% NaOCl/10% SA/17% EDTA 12.52 ± 2.48	The data were normally distributed. Hence, one-way Analysis of variance (ANOVA) was used to test the difference among groups at a 95% confidence interval. Post hoc Tukey’s test was performed to compare the difference among the groups.	Effective
Stevens, 2014 USA [29]	dentinal root	μTBS Test	1. Negative control with Variolink (V) II 18.8 ± 4.2; Multilink (M) 29.1 ± 7.1; Clearfil Esthetic Cement EX (CECEX) 20.7 ± 4.9; SpeedCEM (SCEM) 17.8 ± 4.2; Clearfil SA Cement (CSAC)7.2 ± 2.8 2.6% NaOCl 20 min: V II 24.0 ± 6.7; M 34.1 ± 6.1; CECEX20.7 ± 6.8; SCEM 00.0 ± 0.0; CSAC 00.1 ± 0.1 3.SA 5 s: SCEM 8.5 ± 2.6; CSAC 4.3 ± 2.0 4.SA 1 min; SCEM 12.1 ± 3.2; CSAC SA Cement 4.8 ± 10.	Statistical analysis of data was performed with analysis of variance and Tukey post hoc test. The significance level was set at *p* < 0.05.	Neutral
Wang et al., 2019 China [33]	dentinal coronal surface	μTBS test	Blank control (deionized water) 48.71 Negative control 24.46 (5.25% NaOCl 30 min) PA 5%/1 min 26.62 PA 5%/5 min 35.88 PA 5%/10 min 39.11 PA 10%/1 min 31.25 PA 10%/5 min 41.39 PA 10%/10 min 46.98 PA 15%/1 min 36.50 PA 15%/5 min 48.21 PA 15%/10 min 53.16	microTBS data were analysed using one-way ANOVA and the Write Student–Newman–Keuls test. The confidence interval test was performed to analyse the recovery effect of PA on bond strength to NaOCl-treated dentine. The chi-squared test was used to analyse failure mode distribution. The statistical significance level was set at α = 0.05.	Effective
Weston et al., 2007 USA [30]	dentinal root	μTBS test	1.0.9% NaCl 10 min 23.3 ± 4.9 2. 5.25% NaOCl 10 min 8.3 ± 3.5 3. 10% SA 10 min 29.1 ± 8.9 4. 10% SA 3 min 22.0 ± 7.6 5. 10% SA 1 min 23.8 ± 5.1 6. 20% SA 1 min 23.6 ± 7.3	Three-way analysis of variance was performed using surface treatments (NaCl, NaOCl, or NaOCl/SA) as 1 factor, treatment times and concentration of sodium ascorbate (10% for 10 min, 10% for 1 min, 10% for 3 min, or 20% for 1 min) as the second factor, and location (i.e., cervical, middle, or apical third) as the third factor. Multiple comparison post hoc tests were performed using Tukey’s honest significance difference test at α = 0.05.	Effective

## Data Availability

Data sharing not applicable.

## Supplementary Materials

The following supporting information can be downloaded at: https://www.mdpi.com/article/10.3390/ma16062260/s1, Table S1: PRISMA checklist.
